# The Development of Education of Public Health Nurses for Applying Genomics in Preventive Health Care

**DOI:** 10.3389/fgene.2022.849232

**Published:** 2022-04-08

**Authors:** Mari Laaksonen, Elisa Airikkala, Arja Halkoaho

**Affiliations:** School of Social Services and Health Care, Tampere University of Applied Sciences, Tampere, Finland

**Keywords:** education, health promotion, public health nurse, precision public health, genomics

## Abstract

Genetics and genomics have become one of the most important development areas in healthcare. For this reason, it is essential that nursing professionals take their role to offer their skills in implementing genomics in health promotion. The education of public health nurses is taking vital steps in training the health promoters who are able to take the genome-based knowledge into account in precision healthcare. Tampere University of Applied Sciences managed to integrate genomics into the studies of public health nursing. This article describes the process of development and lays emphasis on the importance of genomic education of public health nurses.

## Introduction

Genetics and genomics have become one of the most important areas in healthcare. COVID-19 has most recently shown that when rapid changes are needed, genetics is strongly involved in elucidating the etiology of diseases such as describing the pathophysiological mechanisms or developing therapies and vaccinations (Pairo-Castineira et al., 2021). Genomic-based information is important in healthcare, for example, in more accurate diagnostics of diseases, tests of molecular mechanisms of diseases for personalized drug treatments, pharmacogenetic tests for the anticipation of adverse drug treatment effects, and targeting of disease screenings (Ministry of Social Affairs and Health, 2015). Previously, genetic testing had been focusing on single-gene diseases. Now, there has also been a shift from this reactive testing to proactive testing with genome-based studies adding the prevention of multifactorial diseases to the competence list for professionals (Mills and Haga, 2014). It is obvious that this shift requires a new kind of expertise from healthcare staff. Not only the development of science but also the public awareness of genomic knowledge creates an additional need for education. Citizens have easy access to new genomic information through direct-to-consumer services, and this has created an entirely new need for knowledge for workers in healthcare. Therefore, it is evident that applying genomic information into practice is no longer only specialists’ work (Saleh et al., 2019).

To accomplish these demands, building comprehensive competencies for genomics in nursing is essential. In studies measuring genomic competence of nurses or midwives, the knowledge level has been described to be low to average (Skirton et al., 2012; Calzone et al., 2014; Wright et al., 2019). Nurses have indicated that it is important to become more educated about the genetics of common diseases (Calzone et al., 2014). According to Camak’s (2016) review, nurses are underprepared for incorporating the applications of genetics and genomics to patient care, and they have shown low confidence in delivering genetics (Williams and Dale, 2016), but nurses who had taken genetics courses since licensure reported higher confidence than those who had not attended any genetics education (Calzone et al., 2014). Newcomb et al. (2019) noticed that nurses performed genomic activities rarely in their clinical work. Therefore, it is clear that confidence in skills does not yet exist, and competence must be strengthened within all the nurses at all levels and across all specialties.

Public health nurses (PHNs), working in primary or occupational healthcare are at the forefront of receiving citizens with the genome test results they have purchased. They also discuss with patients about the genomic data effects on patient families, possibilities of receiving secondary test results, and other ethical issues in the process of acquiring informed consent. In addition to this, PHNs are vital in observation of adverse drug reactions in homecare. However, Goda et al. (2019) reported that public health nurses encountered difficulties in recognizing the genetic issues and were not conscious of them while providing their professional services. In a study of Kawasaki et al. (2021), PHNs benefited from education and achieved deeper understanding of genomics and their own role in patient education with genomic issues.

The long-term goal of genomics is said to be a reduction of health inequalities (Ministry of Social Affairs and Health, 2015). Public health nurses have a significant role in achieving this. Their competencies include implementing strategies to reduce health inequalities in population and at the individual intervention level (Mabhala, 2015). Although activities at the community level are not performed as often as at the individual level in PHNs’ daily work, both are core responsibilities (Haron et al., 2019). At the individual level, mitigation of the impact of genetic risk and lifestyle counseling are a part of the preventive services delivered by public health nurses.

It is recognized in the Finnish National Genome Strategy (Ministry of Social Affairs and Health, 2015) that the capacity of healthcare professionals to use and implement genome-based information should be strengthened. Nurses are vital professionals in implementing information and achieving the goals of genomic information in their day-to-day work with patients (Whitley et al., 2020). Nursing is the largest healthcare profession. There are about 60,194 nurses, 9,301 public health nurses, and 2 417 midwives in Finland working as employees and entrepreneurs (OSF, 2019). The number of nursing students graduating and entering working life each year is about 4, 000 in Finland. In 2020, this consisted of 3, 076 nurses, 669 public health nurses, and 36 midwives (OSF, 2021). Such a large workforce should not be overlooked. Thus, attitude, perception, confidence, and knowledge of genomics of all nurses are essential factors when integrating genomic applications into practice.

In the last decades, nursing education contained little of genetics (Calzone et al., 2014; Calzone et al., 2016; Wright et al., 2019), and most of the current nursing workforce graduated before this genomic era. Effective education is, therefore, needed both at the undergraduate level and from the perspective of continuing education (Whitley et al., 2020).

The lack of published core genomic competencies of nursing at the national level in Finland led to the development of education. Tampere University of Applied Sciences has been profiled for the extensive use of gene and genome information in healthcare. The PROFITU strategic project (years 2019–2021), funded by the Ministry of Education and Culture, has developed curricula in the fields of healthcare, both bachelor and master levels, by adding studies related to genetics and genomics into the curricula. In addition, continuing education has been planned for different target groups, which has increased due to the need of working life.

This article describes the process of development and lays emphasis on the importance of genomic education of public health nurses.

### The Need for Genomic Competence in Public Health Nurses’ Services

Mills & Haga (2014) highlighted the difference between traditional genetic counseling and genomic counseling. Genomic counseling applies to diverse types of diseases. In addition, the purpose of the test, intervention, and clinical utility, and the access to the test, differ from the traditional situation of a genetic test. Mills and Haga (2014) suggest expanding the role of genetic counselors as a solution to increasing the need of proactive risk reduction intervention and preventive counseling. On the other hand, it is conceivable that public health nurses and other health promotion experts should extend their competence to genomic counseling (McBride, 2008; Terry, 2020), in which the case preventive health counseling would continue naturally after testing. These interventions would aim to promote health and prevent diseases with accurate and new methods of health promotion. The methods provided in health counseling would include diverse approaches such as health technologies, group meetings, and individual receptions of patients in healthcare in a way of precision healthcare.

Since genomics is no longer a medical specialty for rare diseases but increasingly related to multifactorial diseases affecting a substantial portion of the population, the need for genomic knowledge is growing among a variety of stakeholders, including nurses (Syurina et al., 2011). Advances in genome research, particularly in the risk assessment of multifactorial diseases, increase the need for genomic training for public health nurses. PHNs in Finland work typically in services governed by the Health Care Act to support health and welfare promotion among the residents and prevent illnesses. In addition to this, the provision of health counseling should be included in all the health care services (Health Care Act, 1326/2010, 2010). PHNs work in these services, for example, in maternity and child health clinic services, in school-based health care, in student health care, in occupational health care, and in home care, promoting health and preventing diseases with all age groups. Health counseling preventing multifactorial diseases is emphasized at the receptions of public health nurses. Therefore, the genome-related information should be included in the competence of the PHNs. PHNs have possibilities to carry out early interventions and targeted screenings, and genomics can help identify the risks of multifactorial, common complex diseases at an early stage. In addition to that, PHNs can provide lifestyle guidance and counseling which would complete the intervention. Studies (Godino et al., 2016; Hollands et al., 2016) have shown that the genome-based information alone does not change lifestyle into a healthier one. In turn, the genome-based data with support of PHN could more likely motivate clients to make permanent lifestyle changes.

### The Development Process of the Curriculum of Public Health Nurses

**FIGURE 1:**
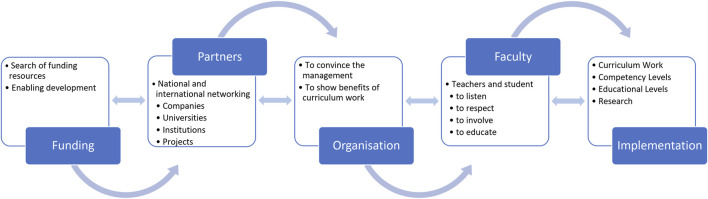
Roadmap of the process of implementing G/G in nursing at TAMK.

This article describes a direct approach of an education organization that was used in creation of curricula for nurses and public health nurses in Finland. The process of implementing genetics and genomics into the curricula is illustrated in Figure 1.

This work was made possible through the PROFITU -project, funding of the Finnish Ministry of Education and Culture. The faculty of nursing in Tampere University of Applied Sciences (TAMK) understood the need for genome education in nursing through rapidly developed genomic sequencing, its transitioning into clinical practice, and implementation into healthcare systems. The keypersons in the faculty observed the development of genomic science and its integration into education and training, for example, in England, where a big volume national program delivered 700 person-years of education to increase the competence of workforce (Stark et al., 2019). Collaboration with national and international partners was relevant to get a wide view of genetics and genomics. Networks were created extensively through ethics, technology companies, geneticists, biobanks, institutions, and experts of genomic nursing.

In addition to national legislation and EU directive, the competence requirements in nursing in Finland are developed regularly in cooperation with the universities of applied sciences and the Ministry of Education and Culture. The developed competence requirements in Finland can be used as a basis for planning the curricula in autonomous universities of Applied Sciences (Savonia, 2020). However, the latest descriptions made in 2019 do not include the genome-related topics at all (Savonia 2020). The Finland National Genome Strategy 2015–2020, describing the proposal of future infrastructure, is the only guideline in Finland to mention education to include genomics (Ministry of Social Affairs and Health, 2015). The guideline of Consensus Panel (2009) for genetic and genomic nursing was a supporting model for the integration of genetics and genomics into the curricula in TAMK. For the curriculum work, the project team asked help from the faculty departments to create objectives for teaching different nursing sectors from the aspect of genetics and genomics. This way, the faculty was engaged, and the teachers had the opportunity to participate, learn, and influence.

Implementing genomic education for the training was seen as a process proposed by Rogers (2003): The new ideas disseminate through diffusion stages widely in faculty. First, the individuals excited by genomics were invited to learn and communicate with the rest of the faculty through various channels to adopt the innovation. Several seminars and mandatory education sessions were organized. Articles and blogs were published to educate and increase enthusiasm within the faculty. The faculty was invited to a workshop to discuss the ethical aspects of genomics. This was an important step to commit the teachers. The acceptance of the fact that the individuals in the educational organization will adopt or accept an innovation, such as genomics in nursing, at different time points (Rogers, 2003), was essential.

To raise public awareness of genomic literacy and genomic health prevention opportunities, new partnerships between education, healthcare, and government organizations are required, not forgetting the media and citizens (Hurle et al., 2013). Education organizations collaborating with the other genomics-related organizations were important in the PROFITU project. The collaboration with Finnish Clinical Biobank Tampere (Finnish Clinical Biobank Tampere, 2021) and the Finnish Institute for Health and Welfare (2021) was essential. Student involvement for the developing process in every stage was also important: by participating in biobank week with Biobank Tampere, participating in lectures by the national and international experts alongside teachers, piloting *The Basics of Genetic Nursing* course, by doing genomics-related bachelor and master theses, and other development works. One example is student development work, in which THL, the Finnish Institute for Health and Welfare, provided a script for the animation of polygenic risk score, and the PHN students implemented it as an animation (Supplementary File 1).

The outcome objective of the development process was that all nurses graduating from TAMK will have a foundation of knowledge in basic human genetics and genomics and current applications to nursing practice as recommended for the workforce competencies by Genomic Nursing Competency Implementation Strategic Plan (2014). ECTS credits (The European Credit Transfer System) are used by universities in Europe in nursing programs (European Commission, 2022). The degree program in Public Health Care (240 credits) in Finland includes Nursing Care studies (180 credits), where genomic nursing competence is integrated in the curriculum because of the project (Table 1). The integration of genomics within the courses has been considered to be functioning well, although it required a lot of collaboration between those who are teaching, in addition increasingly in the nursing faculties had to acquire knowledge about genomics for their own field (Daack-Hirsch et al., 2013). TAMK included a new 2-credit standalone course, *The Basics of Genetic Nursing*, to the curriculum starting in autumn 2021, and integrated genomics into the existing courses according to the content. Genomics was integrated widely, for example, *Internal medicine*, *Oncology*, *Nursing at a Health and Social Services Center*, and *Nursing Care for the Elderly*. After nursing care studies, public health care students increased their genomic knowledge, for example, with fetal screening, polygenic risk score in multifactorial diseases, counseling skills, and ethics as part of *Basics of Public Health Nursing*, *Adult Health Care*, *Pregnant and Neonatal Family,* and *Child and Family Health Care*.

**TABLE 1:** Genetic and genomic content integrated into the degree program in public health care.

Degree programme in public health care 240 ECTS Credits, 4 years	
Nursing care studies 180 cr	Public health care studies 60 cr
The Basics of Genetic Nursing, 2 cr standalone course	Genetics and genomics content integrated to
Genetics and genomics content integrated to	• Basics of Public Health Nursing
• Internal Medicine	• Public Health Nursing: Adult Health Care
• Oncology	• Public Health Nursing: Pregnant and Neonatal Family
• Surgery	• Public Health Nursing: Child and Family Health Care
• Nursing Care of Internal Medicine and Palliative Care	—
• Nursing of Children and Young People and Nursing of the Disabled	—
• Mental Health and Substance Abuse Nursing	—
• Nursing at a Health and Social Services Center	—
• Nursing Care for the Elderly	—

## Discussion

Health promotion is changing rapidly due to genomic information. Nonetheless, there is a debate about whether genomics can bring anything new to health promotion. What is new about genomics to promote community health and whether diseases are overemphasized at the expense of health? In addition, the technology associated with the genome has also been questioned. (McBride, 2008; Terry, 2020). Thus, genomic science is developing and making the implementation challenging. (Whitley et al., 2020). It must be noted that the change is taking place and that it must also be prepared from an educational point of view. We agree that genomics will come to play a vital role in precision public health and health promotion, but it also requires concurrent knowledge on the basic variables of health promotion such as determinants of health, environmental factors, and health education methods. (Terry, 2020). This means new responsibilities to the public health nurses. The education of PHNs covers the basic elements of health promotion in different age groups. As a result, they play a key role when implementing genomic information into preventive precision public health.

As science takes steps forward in work life, education should be at the forefront of this development and to prepare nurses to face future demands. Nurses often experience considerable strain at work with demands from new competencies (Hasson & Arnetz, 2008). Education can transform requirements into a resource to experience new aspects, such as genomics, that stimulates professional growth and development to achieve work goals more effectively, and to provide high-quality nursing care as Broetje and Jenny (2020) describe. We agree with the consensus panel when they emphasized that in genomics, it is essential to define what a nurse (all academic levels, roles, and clinical specialties) is required to know about genomics to achieve competency (Consensus Panel, 2009). However, the challenge is that there is a gap in knowledge among the healthcare professionals and many competing priorities in nursing (Whitley et al., 2020). To fulfill these challenges, there is a need for close cooperation with the various stakeholders. Educational organizations are responsible for this development work together with working life. It is important to create a new type of training that increases the skills of those already employed. In an area as renewed as the genome is, it is vital to work closely with the scientific community as well. In this way, educational organizations are always able to be at the forefront of new knowledge and develop confidence in knowledge of and communication skills about genomics. This might allow nurses personal investment to drive learning new skills. (Whitley et al., 2020).

In this educational development, TAMK was a pioneer, both nationally and internationally. National development can often be slow, and therefore it is important that TAMK is able to develop new types of expertise in healthcare. Large-scale integration requires commitment from the education organization and its management. Adding something new, such as comprehensive content, takes time, investment, and administrative/management support. Funding was also crucial in enabling us to train teachers on new knowledge. Finnish Ministry of Education and Culture partially funded the TAMK PROFITU–project to profile new competencies to general and public health nursing as a pioneer education organization. However, the following years really show how successfully genomics was integrated when the new curricula are in place and evaluated. In addition, the courses should be constantly evolving in-line with the developments in genomic research and genomic nursing practical needs. Development work with international partners will continue, for example, as a virtual student exchange at the master level. In addition, new Erasmus + funding will create new possibilities internationally.

Genomic nursing is still looking for its direction and its operating environments in Finland. The nurses are experts in their fields, so their expertise in genomic knowledge should be visible in nursing activities too. The utilization of genomic knowledge requires multidisciplinary collaboration (Syurina et al., 2011), and nurses have their part and role to play in that collaboration. No one is going to give that role to nurses from the outside. In addition, the development of genomics in nursing does not mean passively waiting for something to just happen or waiting for someone else to say or determine what genomic nursing expertise is. Nursing managers, educators, and researchers should take an active role so that genomic competencies can be increased in nursing care.

## Data Availability

The raw data supporting the conclusions of this article will be made available by the authors, without undue reservation.
